# Tympanometric Findings among Children with Adenoid Hypertrophy in Port Harcourt, Nigeria

**DOI:** 10.1155/2016/1276543

**Published:** 2016-08-02

**Authors:** Chibuike Nwosu, Mathilda Uju Ibekwe, Lucky Obukowho Onotai

**Affiliations:** Department of Ear, Nose and Throat Surgery, University of Port Harcourt Teaching Hospital, Port Harcourt, Nigeria

## Abstract

*Introduction*. Adenoid hypertrophy (AH) is a common childhood disorder. Adenoid plays a significant role in the pathogenesis of otitis media with effusion (OME). The aim of this study is to critically appraise the tympanometric finding among children with adenoid hypertrophy in Port Harcourt, Nigeria.* Methodology*. A Prospective, controlled study carried out among newly diagnosed cases of adenoid hypertrophy at the ENT clinic of the UPTH, between November 2014 and June 2015. Tympanometry was done on each child and each ear was considerably studied as a single entity. Types B and C tympanograms were used as indicators of OME. Data was collected and analyzed using SPSS version 20.* Results*. Sixty-eight cases of adenoid hypertrophy were seen within the study period and 136 ears were studied. Forty (29.4%) ears had type B tympanogram, while 36 (26.5%) ears had type C. The incidence of OME was 55.9%; there were 12 (17.6%) unilateral OME, while bilateral OME was 32 (47.1%). Grade 3 AH was prevalent and was statistically significant with the OME.* Conclusion*. This study had shown adenoidal hypertrophy as a significant risk factor for OME in children. There was more bilateral OME than unilateral. The more severe grade of AH was more prevalent and it was shown to be statistically significant with OME, thus being a significant risk factor for OME in children. This establishes the need for prompt hearing evaluation and management.

## 1. Introduction

Adenoid hypertrophy is a common childhood disorder [1]. It plays a significant role in the pathogenesis of OME which is the commonest cause of hearing impairment in young children [1, 2]. Thus, it predisposes to delayed speech, poor academic and language development [3, 4]. The adenoid forms the uppermost part of the ring of lymphoid tissues in the pharynx (Waldeyer's ring). It is located in the superior posterior wall of the nasopharynx adjacent to the choana and Eustachian tube (ET) opening.

The size of adenoids varies from child to child and also in the same individual as the child grows. In general, it attains maximum size between the ages of 3 and 7 years and then regresses [5]. However, there is significant growth of the soft tissue of the nasopharynx between the age of 3 and the age of 5 years, which leads to the narrowing of the nasopharyngeal airway [6]. Subsequently, the growth of the nasopharynx increases while the soft tissues remain relatively unchanged and thus the airway increases [7].

Adenoids may become chronically infected and act as a reservoir in upper respiratory infections with resultant oedema and obstruction of the nasopharyngeal end of the Eustachian tube (ET) [8–10]. Enlarged adenoids can also lead to mechanical obstruction of the ET, leading to absorption of air and negative intratympanic pressure [11, 12]. Chronic infection of the adenoid tissue can cause epithelial metaplasia and connective tissue fibrosis which impede the function of the cilia and adenoid tissue in clearing infection [13].

Studies have shown that there is an increase in number of mast cells and allergic mediators in adenoid tissues which are capable of binding immunoglobulin E (IgE) and releasing histamines and other inflammatory mediators following exposure to allergens [14]. The mediators released influence the mucociliary transport time, modify the ciliary function and structure, and increase the secretory activity of the mucosal cells of the middle ear [15].

The resultant effects show that ET dysfunction is the most important factor in the pathogenesis of otitis media with effusion [16]. Obstruction of the ET leads to increased middle ear pressure, while there is influx of bacteria and viruses from the nasopharynx following adenoidal infection [17]. This causes mucosal oedema, inflammation, and increased secretory activity of the middle ear mucosa, leading to formation of effusion [17].

In our setting there is paucity of information on tympanometric findings of children with adenoidal hypertrophy. Therefore, we decided to carry out this study to critically appraise the tympanometric findings among children with adenoid hypertrophy in Port Harcourt, Nigeria.

## 2. Methodology

This is a prospective case-control study carried out among newly diagnosed cases of adenoid hypertrophy (AH) at the Ear Nose and Throat (ENT) Clinic of University of Port Harcourt Teaching Hospital (UPTH), Port Harcourt, Nigeria.

The study was carried out between November 2014 and June 2015 and it included all new cases with clinical and radiologic features of AH. Those excluded are patients with previous adenoidectomy, cerebral palsy, generic syndrome, ear discharge, Tympanic membrane perforation, cleft palate, and congenital ear deformities.

Ethical clearance was given by our institution and informed consent was taken from the parents/guardian of all recruited patients/control cases. A complete ENT and physical examination was carried out for all patients.

Only the new patients that had features of AH had plain radiographs of the postnasal space, and adenoid nasopharyngeal ratio (ANR) was measured as proposed by Fajioka et al. [18] and was graded using Sade method (Grades 0, I, II, and III) [19].

The control group was recruited using consecutive sampling from students of Celia International Primary and Nursery School, Port Harcourt, and was matched for age and sex. All had same exclusion criteria with case group and also excluded those with symptoms suggestive of AH. Plain radiograph of the postnasal space was not done for this group.

Tympanometry was done using Auto Tymp 262 Welch Allyn, USA, for both case and control and each ear was studied as a single entity. Types B and C were used as indicator of OME. The data was collected in a Proforma and analyzed using SPSS version 20. *p* < 0.005 was considered significant and confidence interval was set at 95%.

## 3. Results

Sixty-eight cases of AH were seen within the study period. They were all within the ages of 1–10 years. This is shown in Table 1. The mean age was 4.1 and modal age was 2 years. There was male preponderance (M : F = 1.4 : 1).

**Figure 1:**
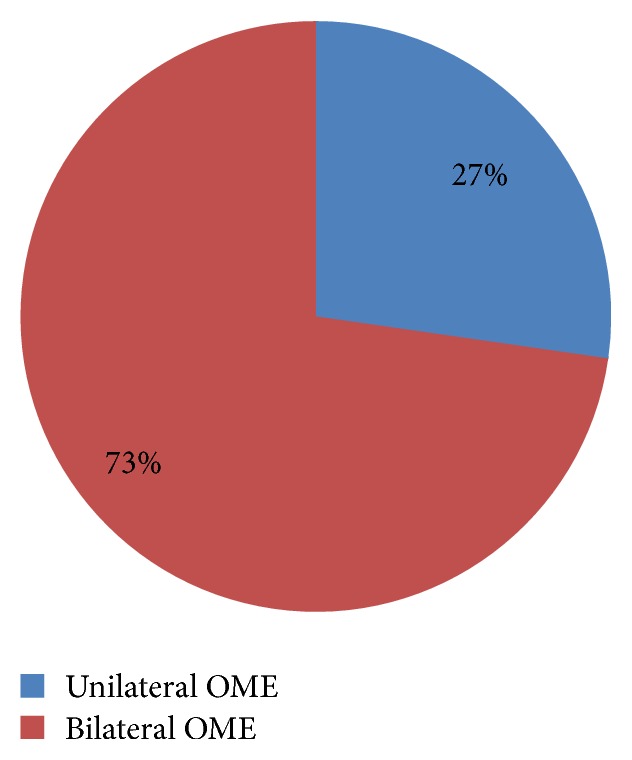
Laterality of OME.

**Figure 2:**
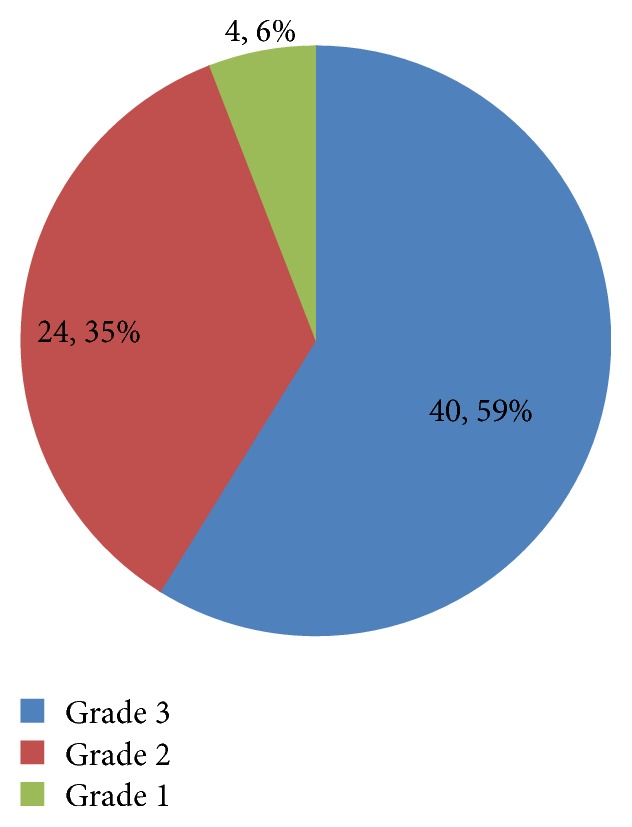
Grades of adenoid hypertrophy. 4, 24, and 40 represent the numbers in terms of frequencies, while 6, 35, and 59 represent the percentages.

The incidence of type B tympanogram was 29.4%, while type C was 26.5% (Table 2). In the control group, type B was 3.7% while type C was 11.8 (Table 3). The incidence of OME in the case group was 55.9% while in the control group it was 15.5% (Table 4) showing a 4-fold increase. Patients with Grade 3 AH were shown to be statistically significant with the occurrence of OME (*p* < 0.05). This is shown in Table 5. There were more bilateral cases of OME (73%) as shown in Figure 1. Among all the patients with AH, those with Grade 3 hypertrophy were more prevalent (Figure 2).

## 4. Discussion

The incidence of OME among patients with AH was 55.9% in this study with more type B (29.4%) than type C (26.5%). When compared with control, there was about a 4-fold increase in incidence of OME. This establishes significance of AH as a risk factor in the pathogenesis of OME. This is similar to the findings reported in Enugu [20] by Orji et al. with incidence of 35% using only type B and also a 7-fold increase in incidence when compared with the control which was statistically significant. Also, there was a similar report in Kenyatta National Hospital [21], among children aged 1 to 4 years with AH at the out-patient clinic with prevalence of 67.3% using both type B and type C as indicators. The study also shows an 11-fold increase in the prevalence of OME when compared with the control group.

There was higher proportion of type B tympanogram than type C in this study. This means that middle ear effusion occurs more than ET dysfunction in patient with AH, which is more associated with severe hearing impairment [22]. This finding was similar to the finding in Kenya with prevalence of type B (67.3%) being about 12-fold higher than type C (5.8%). This reveals the need for prompt hearing assessment and management in those with AH [21].

There were more bilateral cases of OME than unilateral. Generally, bilateral hearing impairment causes more sequelae than unilateral hearing impairment for obvious reasons [23, 24], hence, establishing that patients with AH are more at risk of having sequelae from OME associated with hearing impairment.

All the children in this study had ANR measured. Most (59%) of the patients had grade 3 AH which is synonymous with severe disease. This study had showed significant association between Grade 3 AH and OME when compared with other grades of AH. This report was similar to the study by Hibbert and Stell [25] and with the study in Enugu [20], which also reported positive correlation between the degrees of AH and OME. This shows that the increasing grades of AH are an important predictor in the establishment of OME in patients with AH. Although different objective modalities have been proposed for the diagnosis of adenoid hypertrophy (including mirror examination, palpation, lateral neck radiography, or nasal endoscopy), the role of each of these diagnostic methods is still controversial, and currently there is no comprehensive guideline for assessing adenoidal enlargement [26]. Plain radiographic assessment of the postnasal space of children with features of AH is routine in our centre while the use of fibre-optic endoscope is still undeveloped.

## 5. Conclusion

This study had shown a high incidence of OME among patients with AH with type B tympanogram being more common. There was more bilateral OME than unilateral. The more severe grade of AH was more prevalent and it was shown to be statistically significant with OME, thus being a significant risk factor for OME in children. This establishes the need for prompt hearing evaluation and management.

## Recommendation

(1) Children with AH should be properly screened for OME and proper management instituted to prevent the occurrence of the sequelae. (2) There should be proper enlightenment among medical practitioners especially those practicing in the rural regions in the evaluation of patients with AH and early detention of OME.

## Figures and Tables

**Table 1:** Age range of the patients.

Age range (years)	Frequency	Percentage
<2	8	11.8
2–4	36	52.9
5–7	16	23.5
8–10	8	11.8

*Total *	68	100

**Table 2:** Tympanometric findings in the patients with AH.

Tympanometry	Left ear		Right ear		Both ears	
	Frequency	%	Frequency	%	Frequency	%
A	32	47.1	28	41.2	60	44.1
B	12	17.6	28	41.2	40	29.4
C	24	35.3	12	17.6	36	26.5

*Total*	68	100	68	100	136	100

**Table 3:** Comparing tympanogram in the case and control group.

Tympanometry	Case group		Control group	
	Frequency	Percentage	Frequency	Percentage
A	60	44.1	115	84.5
B	40	29.4	5	3.7
C	36	26.5	16	11.8

*Total*	136	100	136	100

**Table 4:** Incidence of OME.

	Case group		Control group	
	Frequency	Percentage	Frequency	Percentage
OME	76	55.9	21	15.5
No OME	60	44.1	115	84.5

*Total*	136	100	136	100

**Table 5:** Association between OME and Grade 3 AH.

	Adenoid Grade 3		df	*χ* ^2^	*p* value
	Yes	No			
OME	64	12	1	5.8165	0.02
No OME	16	44			

*Total*	80	56			

## References

[1] Bluestone C. D. (1975). Obstructive adenoids in relation to otitis media. *Annals of Otology, Rhinology and Laryngology*.

[2] Yasan H., Dogru H., Tuz M. (2003). Otitis media with effusion and histopathologic properties of adenoid tissue. *International Journal of Pediatric Otorhinolaryngology*.

[3] Pelton S. I. (1996). New concepts in the pathophysiology and management of middle ear disease in childhood. *Drugs*.

[4] Roberts J. E., Burchinal M. R., Jackson S. C. (2000). Otitis media in early childhood in relation to preschool language and school readiness skills among African American children. *Pediatrics*.

[5] Jalisi M., Jazbi B. Chronic middle ear effusion. Current problems in otorhinolaryngology.

[6] Acharya K., Bhusal C. L., Guragain R. P. (2010). Endoscopic grading of adenoid in otitis media with effusion. *Journal of the Nepal Medical Association*.

[7] Jeans W. D., Fernando D. C. J., Maw A. R., Leighton B. C. (1981). A longitudinal study of the growth of the nasopharynx and its contents in normal children. *British Journal of Radiology*.

[8] Tomonaga K., Kurono Y., Chaen T., Mogi G. (1989). Adenoids and otitis media with effusion: nasopharyngeal flora. *American Journal of Otolaryngology—Head and Neck Medicine and Surgery*.

[9] Gates G. A. (1994). Adenoidectomy for otitis media with effusion. *Annals of Otology, Rhinology & Laryngology*.

[10] Dhingra P. L. (2009). Eustachian tube and its disorders. *Disease of the Ear Nose and Throat*.

[11] Bluestone C. D., Cantekin E. I., Beery Q. C., Paradise J. L. (1975). Eustachian tube ventilatory function in relation to cleft palate. *Annals of Otology, Rhinology and Laryngology*.

[12] Di Francesco R., Paulucci B., Nery C., Bento R. F. (2008). Craniofacial morphology and otitis media with effusion in children. *International Journal of Pediatric Otorhinolaryngology*.

[13] Kiroğlu M. M., Özbilgin K., Aydoğan B. (1998). Adenoids and otitis media with effusion: a morphological study. *American Journal of Otolaryngology—Head and Neck Medicine and Surgery*.

[14] Berger G., Ophir D. (1994). Possible role of adenoid mast cells in the pathogenesis of secretory otitis media. *Annals of Otology, Rhinology and Laryngology*.

[15] Passali D., Passali G. C., Lauriello M., Romano A., Bellussi L., Passali F. M. (2014). Nasal allergy and Otitis media: a real correlation?. *Sultan Qaboos University Medical Journal*.

[16] Xia Z., Wang Z., Cui L., Wei C., Liu Y., Huang F. (2014). The observational and analysis of the function and morphology of the ET in OME and chronic rhinosinusitis in children. *Lin Chung Er Bi Yan Hou Tou Jing Wai Ke Za Zhi*.

[17] Margaret A. K., Adriane D. L., Byron J. B., Jonas T. J., Shawn D. N. (2006). Epidemiology of otitis media with effusion. *Head and Neck Surgery-Otolaryngology*.

[18] Fujioka M., Young L. W., Girdany B. R. (1979). Radiographic evaluation of adenoidal size in children: adenoidal-nasopharyngeal ratio. *American Journal of Roentgenology*.

[19] Sade J. (1979). *Secretory Otitis Media and Its Sequel*.

[20] Orji F. T., Okolugbo N. E., Ezeanolue B. C. (2010). The role of adenoidal obstruction in the pathogenesis of otitis media with effusion in Nigerian children. *Nigerian Journal of Medicine*.

[21] Mwaniki K. A. (2015). *Prevalence of otitis media with effusion in children with obstructive adenoid tissue compared with normal control at the Kenyatta National Hospital. A dissertation in partial fulfillment of the requirement for the degree of masters of medicine at the university of Nairobi, Kenya [M.S. thesis]*.

[22] Li Y., Hunter L. L., Margolis R. H. (1999). Prospective study of tympanic membrane retraction, hearing loss, and multifrequency tympanometry. *Otolaryngology—Head and Neck Surgery*.

[23] Silva P. A., Kirkland C., Simpson A., Stewart I. A., Williams S. M. (1982). Some developmental and behavioral problems associated with bilateral otitis media with effusion. *Journal of Learning Disabilities*.

[24] Gravel J. S., Wallace I. F. (2000). Effects of otitis media with effusion on hearing in the first 3 years of life. *Journal of Speech, Language, and Hearing Research*.

[25] Hibbert J., Stell P. M. (1982). The role of enlarged adenoids in the aetiology of serous otitis media. *Clinical Otolaryngology and Allied Sciences*.

[26] Lertsburapa K., Schroeder J. W., Sullivan C. (2010). Assessment of adenoid size: a comparison of lateral radiographic measurements, radiologist assessment, and nasal endoscopy. *International Journal of Pediatric Otorhinolaryngology*.

